# EEG-Based Classification of Internally- and Externally-Directed Attention in an Augmented Reality Paradigm

**DOI:** 10.3389/fnhum.2019.00348

**Published:** 2019-10-09

**Authors:** Lisa-Marie Vortmann, Felix Kroll, Felix Putze

**Affiliations:** Cognitive Systems Lab, Department of Mathematics and Computer Science, University of Bremen, Bremen, Germany

**Keywords:** internal attention, external attention, EEG, augmented reality, classification, brain-computer interface

## Abstract

One problem faced in the design of Augmented Reality (AR) applications is the interference of virtually displayed objects in the user's visual field, with the current attentional focus of the user. Newly generated content can disrupt internal thought processes. If we can detect such internally-directed attention periods, the interruption could either be avoided or even used intentionally. In this work, we designed a special alignment task in AR with two conditions: one with externally-directed attention and one with internally-directed attention. Apart from the direction of attention, the two tasks were identical. During the experiment, we performed a 16-channel EEG recording, which was then used for a binary classification task. Based on selected band power features, we trained a Linear Discriminant Analysis classifier to predict the label for a 13-s window of each trial. Parameter selection, as well as the training of the classifier, were done in a person-dependent manner in a 5-fold cross-validation on the training data. We achieved an average score of approximately 85.37% accuracy on the test data (± 11.27%, range = [66.7%, 100%], 6 participants > 90%, 3 participants = 100%). Our results show that it is possible to discriminate the two states with simple machine learning mechanisms. The analysis of additionally collected data dispels doubts that we classified the difference in movement speed or task load. We conclude that a real-time assessment of internal and external attention in an AR setting in general will be possible.

## 1. Introduction

Daily life includes many situations where we find ourselves lost in thought or thinking about something while we were supposed to pay attention to someone or something in our outside world: a speaker, a teacher, a movie, or a colleague talking to us. At some point, we may realize that we do not remember anything they said or did the last minutes, our attention was directed inside ourselves. We suppressed the external influences and our sensory input to a certain degree, to be able to concentrate on internal processes and thoughts. “External attention refers to the selection and modulation of sensory information, as it initially comes into the mind, generally in a modality-specific representation and often with episodic tags for spatial locations and points in time. (…) Internal attention refers to the selection and modulation of internally generated information, such as the contents of working memory, long-term memory, task sets, or response selection” (Chun et al., 2011). We might consciously start an internally directed attention period because we want to think about something, or we do not even realize our mind has been wandering. Sometimes, we cannot even tell how long our mind has been fixated on something other than it was supposed to. This difficulty in reporting one's direction of attention in many situations makes it hard to judge, understand, and scientifically work with one's attentional state. However, an estimation of the attentional state of a person might be helpful in several applications.

With Augmented Reality (AR) becoming more popular, the industry is facing the challenge of making it as intuitive, interactive, and unobtrusive as possible, as this would allow for more application scenarios. One of the problems is the interference of virtually displayed objects with the current attentional focus of the user. Instead of being a helpful or enjoyable addition to the visual field, the projection can be a distraction. Thus, knowing the attentional state of a person would greatly improve the applicability of AR devices and even open up new possible use cases, specifically designed to detect, react to, and work with this attentional state. It follows that measuring attention independently and implicitly, without the necessity of participant intention, is a main goal in our research.

Brain-Computer Interfaces (BCI) offer physiological information about the user's mental state to the technical system. Sensing techniques, such as Electroencephalography (EEG), have improved drastically over the past decades and make reliable capturing of brain activation possible. This brain activation has to be decoded and therefore, “translated” into actual thought patterns and behavioral correlates before this information can be used to adapt a system's state to a user's state. As an example scenario, one can imagine a system that monitors the attention of a person driving a car. Once the BCI detects a period of internally directed attention, the driver can be reminded to focus on the road ahead instead.

In this research project, we explored the possibility of decoding the attentional state of the user during an AR paradigm from their EEG data. Our goal was to classify the attentional state regarding internally or externally directed attention through machine learning algorithms, using automatic, individual feature selection. Data accumulation was performed in 90-min sessions in the laboratories of the Cognitive Systems Lab. For this purpose, we designed an experimental paradigm consisting of “internal” and “external” trials, which refers to the direction of the user's attention. These two conditions are conceptually very similar to assure that the classified difference regards only the direction of attention but not merely the task type. The external tasks were implemented to be displayed in Augmented Reality (AR). This approach was chosen because it adds another dimension to otherwise 2-dimensional computer tasks aiming at improving the similarity to daily, real-world scenarios. Beyond that, the possible improvement of AR applications through such attention measurements that was described earlier motivated this setup.

Based on related work on neurological differences between the two states, we assume enough difference in the recorded EEG for a classification algorithm to discriminating them. For this purpose, we will use a Linear Discriminant Analysis to test the principle hypothesis that an attentional state classification differentiating internal and external attention in an AR setting is possible based on EEG data.

## 2. Related Work

In this section, we review related work on the classification of internally- and externally-directed attention. We found no other work addressing the classification of internal and external attention in an AR task but solely papers with related concepts. First, we briefly show that neurophysiological and behavioral differences have been found that allow for an optimistic perspective on the success of the attempted classification. Second, we introduce research that made use of a user's attentional state in an AR setting, and finally, we address our previous research in this field.

### 2.1. Internally- vs. Externally-Directed Attention

Several studies deal with correlates of internally- and externally-directed attention (or certain aspects thereof) in the EEG signal. Cooper et al. (2003) investigated the well-known effect of increased power in the alpha power band during times of “cognitive idling.” The authors presented evidence that this effect is a frequency marker for active suppression of external stimulus processing during “internally directed attentional tasks.” Benedek et al. (2014) showed differences in the frequency power spectrum in the right parietal region of the brain as markers to differentiate between internal and external attention. Braboszcz and Delorme (2011) analyzed spectral and ERP-related parameters as markers for mind-wandering during an internal attention task. They reported that the parameters they found to correlate with self-reported mind-wandering were similar to parameters correlated with low alertness. Closely related to our research question is the paper by Putze et al. (2016), where they show that EEG data can be classified to discriminate between internal and external attention processes on a single-trial basis in a computer-based experiment using several different tasks for each condition.

### 2.2. Attentional State in AR Settings

Making optimal use of users' attention has been an important goal of research on AR interfaces: Bonanni et al. (2005) used layered interfaces designed according to cuing and search principles of attention theory to reduce the user's cognitive load. Lu et al. (2012) investigate a subtle cuing method to support visual search in AR settings, which are as effective as explicit cuing methods but less distracting. Biocca et al. (2006) introduced the attention funnel technique as a 3D cursor to guide the user's attention toward objects which are completely outside the current visual field. These studies yield important guidelines for building attention-driven AR interfaces. However, most of the proposed AR prototypes do not make use of an online assessment of the user's attentional state. Very recently, researchers in academia and industry showed the feasibility of combining AR and BCI technology: Faller et al. (2017) used an SSVEP-based BCI as a silent and hands-free input channel to a head-mounted AR device. Similarly, Kishore et al. (2014) compared an SSVEP-based BCI as an input mechanism to a head-mounted display for controlling a robot with a gaze-based input mechanism. Mercier-Ganady et al. (2014), in turn, used an AR device to visualize the abstract output of a BCI to the user.

## 3. Methods

We introduce in section 3.1 a novel spatial perspective alignment task which is built upon the Lab-Streaming-Layer to leverage the possibilities of sizeable synchronous data streams in an AR setting (see section 36). The experiment design and technical design enables us to train and evaluate a Linear Discriminant Analysis to discriminate internally and externally directed attention on trigger-bound fixed time windowed data samples (here: trials). The exact methods used during the recording and analysis are described in the following.

### 3.1. Experiment Design

In the work of Putze et al. (2016), a stationary experiment is presented, which is conducted in a closed laboratory environment in front of a regular computer monitor with different tasks for internal and external attention. Hence, the participants have to sit still in front of the monitor and do not move much. The stationary setup reduces unwanted influences but creates a very artificial scenario. Therefore we want to introduce a task which requires the participant to stand and to move for both – an internal condition and an external one. The external condition is specifically designed to run on an AR capable device to leverage the spatial tracking and awareness capabilities as well as the possibilities to fully observe the participant's movements. We use the Microsoft HoloLens, a pair of mixed-reality smart glasses that requires externally directed attention. The internal task is designed in a way which requires the participant to move and use mental rotation. This section will explain the task and its implementation in detail.

#### 3.1.1. Perspective Alignment Task

In order to create a more active task compared to Putze et al. (2016), we developed a spatial perspective alignment task. An additional benefit of this task is that the internal and external condition are very closely related and more similar than the tasks used in the computer-screen based approach. The idea of this task is to complete an object or constellation of objects by changing the perspective – like a solar eclipse that is only visible if the earth is at a specific point on its eclipse or the geometric illusion of an impossible object that can be created with real objects which must be looked at from a specific angle. In AR, this corresponds to tracking a moving object for maintaining the best perspective during the observation.

**Figure 1:**
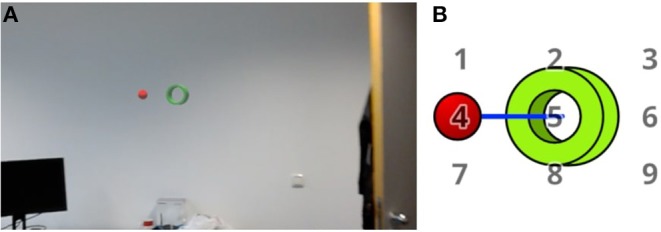
**(A)** The first image shows a screenshot from implementation on the HoloLens. **(B)** The displayed holographic objects: A red sphere and a green tube. Both stay on a fixed axis with a fixed distance marked by the blue line. The pivot of rotation is in the center of the tube. By rotating the object axis around this pivot, the alignment could be changed and therefore force the participant to change their head position accordingly. The displayed numbers are marking the positions to which the red sphere is rotated during the experiment. They are not displayed during the experiment.

**Figure 2:**
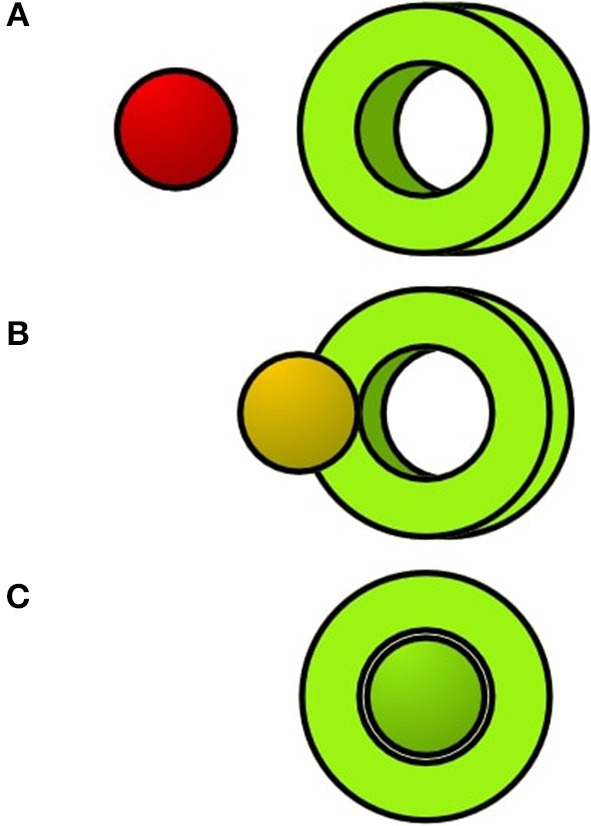
The sphere will change its color if the participant completes the alignment. From **(A–C)** the perspective alignment is getting better.

Our spatial perspective alignment task consists of two objects: A tube and a sphere. Both objects are placed ca. 80 cm from each other away on an axis so that they form a constellation. Figure 1 visualizes the constellation. When looking at those objects from the right perspective, the sphere will be seen inside the hole of the tube – This marks the complete state of the alignment. To better indicate the alignment progression, we colored the object differently and changed the sphere's color to green if it is seen inside the tube. The alignment progression is displayed in Figure 2.

**Figure 3:**
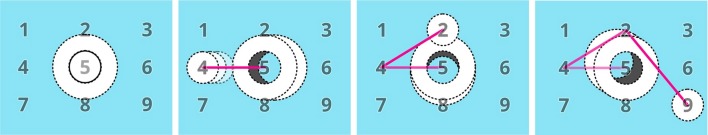
**(Left)** The start of the sequence. **(Right)** The constellation of the sphere and the tube is rotating. The sphere appears to be moving toward number 4 first, then 2 and then 9.

The main task for the participants is to keep the objects perspectively aligned with each other the whole time. To achieve such an alignment, the participants were asked to adjust the position of their upper body in accordance with the current task. We rotate the axis of the constellation in a random but fixed manner. The object movement follows a number grid which is placed in front of the tube, facing the viewer (displayed in Figures 1, 3). If the rotation reaches the goal destination based on a number, it will rotate to the next number. The constant motion forces the participant to move continuously in order to complete the alignment. For our experiment, we choose a 5-s duration for rotating from one number to the next.

The spatial perspective alignment task seems to fulfill many criteria for a task which affords continuous externally directed attention without inducing a high mental load. The participant has to evaluate the state of the alignment continuously by processing their visual feedback and adjust their movement slightly. Since we also aim to produce internally directed attention and movement of the participant, we separated the spatial perspective alignment task in two conditions: One provides direct visual feedback (external), and the other does not (internal).

**Figure 4:**
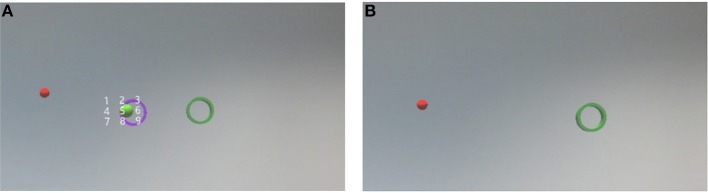
**(A)** Shows a screenshot from the tutorial. During the internal condition, the red sphere and green tube are moved to the side to distort their orientation value for the participant. The example constellation and the number-gird are visible to give an idea about their location, speed, and scale of their movement. **(B)** Shows the internal condition during the experiment. No helper-objects are displayed—the constellation shown during the tutorial and the number-grid is hidden.

- *External Condition:* By choosing a random number between 1 and 9, whereby the last selected number is not included, we define the next number on a number grid to which we rotate the constellation (an exemplary movement of a pre-selected sequence is shown in Figure 3). This procedure will be continued until the end of the experiment on the constellation.
- *Internal Condition:* The internal condition requires the participants to imagine the sphere and the tube and their movement while performing the spacial alignment in their mind. The internal task will induce the same movements of the participant's upper body because, in order to fulfill the alignment, they have to move their upper body and head despite missing visual feedback. To guide the motion of the sphere and tub in the participants' minds, a synthetic voice will announce three numbers before the internal condition starts. These numbers correspond to the number grid and describe the rotation. In Figure 3 an exemplary movement of the sequence 4, 2, 9 is shown. After the announcement of the number sequence, the participants have a short memorization phase before the trial starts, to reduce the memory load during the task. It is important to state that the internal task always disrupts the external one and should not provide visual feedback to the user. During the internal task, the objects displayed in the external task are still present. In order to deliver a distraction-free internal task, we distort the visible (external) constellation by breaking the objects axis and move the sphere and tube to the side of the field of view. We chose this approach because we do not remove the visual feedback completely but only visual clues. An example distortion is shown in Figure 4.

We mark the start an endings of each condition also by two distinct sounds. These are as an orientation for the participant, but not part of the trial. The trial starts afterward.

To give an example of what the participants have to imagine and in what scale, we first display the two objects and a number grid (see Figure 4, left image). During the experiment, these objects will be hidden and used for measuring the misalignment (see Figure 4, right image). The objects and the number grid are in the same scale and holographic position in the room for both the external and the internal condition. Our tutorial consists of four steps in total: First, we show a short example of the external task. Secondly, we show the first version of the internal tutorial described above with all helper-objects visible. It is followed by the same tutorial with a hidden helper-constellation but a still visible grid. The last tutorial is a sample trial identical to the actual experiment.

We decided to run this task by using AR on an AR device like the HoloLens because it provided us the possibility to precisely mark the start and the end of a task, to track the movement of the participant and to measure the misalignment for both conditions. It also made it possible to adjust the height of the experiment for each participant.

#### 3.1.2. Experimental Procedure

**Figure 5:**
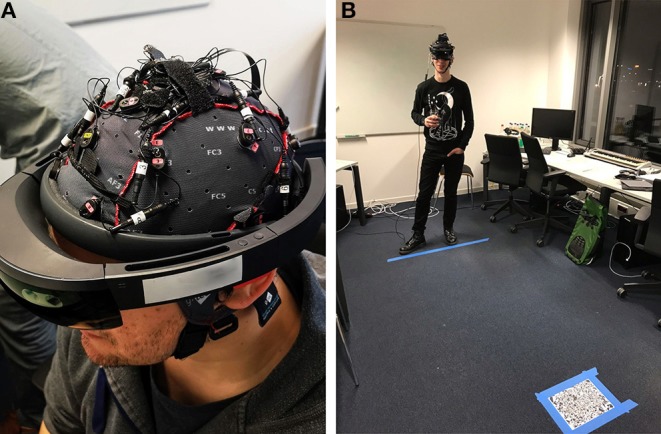
**(A)** The participant wears the EEG-headset and the HoloLens. **(B)** On the floor, the maximum distance to the holographs is marked, and a tracking image for placing the holographic experiment in the room is visible in the front. Written informed consent for the usage of the photos was obtained from identifiable persons.

The HoloLens setup and placement of the participant in the room can be seen in Figure 5. A fixed distance between the subject and the objects was guaranteed through marks on the ground. All described tutorial steps could be repeated as often as necessary until the participant understood the task.

**Figure 6:**
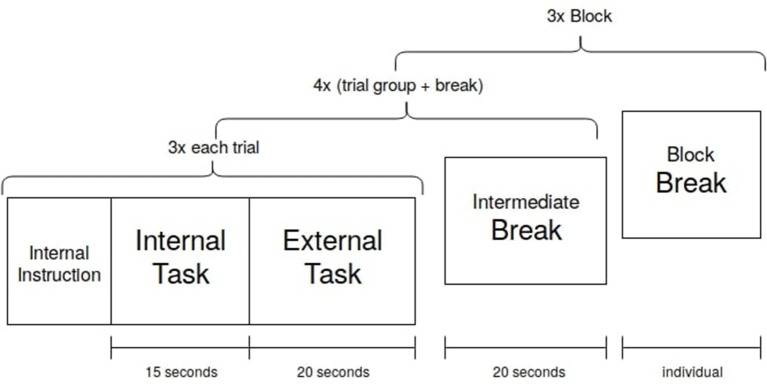
Each trial condition was executed three times before an intermediate break. After four of these trial groups with breaks, a block is finished. The participant chose the length of the block break. Three blocks were recorded.

For the main experiment, the subject starts with an internal task, followed by an external task. Three of these task pairs are followed by a short break with a fixed length. An experimental block ends after the fourth break, thus consists of 12 internal and 12 external trials. The participants performed three blocks with individual breaks in between the blocks. In total, the participants performed 36 internal and 36 external trials in approximately 50 min (Figure 6).

### 3.2. Data Collection

Before the experiment, the participants fill out a demographic and a mind-wandering-related questionnaire. During the main experimental part, besides the logged interaction and movement data, we record EEG and eye tracking data. After completion of the sessions, participants fill out a short questionnaire regarding their perception of the task (an adapted version of the NASA Task Load Index: German language and no category weighting). The total time a participant spent at our lab averaged 2 h with a recording time of 45–60 min. All recordings took place in a normal office at the Cognitive Systems Lab with no special shielding properties, to ensure results that are reproducible in uncontrolled environments.

#### 3.2.1. Participants

Participants were recruited online in a university-independent forum. Fifteen healthy participants (mean age 27.4 ± 10.4; three females) participated in the experiment. All participants had normal or corrected to normal vision. All participants were right-handed, and all but three participants had previously used an Augmented or Virtual Reality Device. We did not restrict the participation in the experiment except for participants with neurological disorders. The local ethics committee approved the study, and written informed consent was obtained from the participants before the conductance of any measurements. Technical problems arose during three of the sessions which led to the complete exclusion of one participant for the further analysis, and two sessions with reduced trial numbers (54 and 48 instead of 72; marked ^*^ in subsequent tables). All the recorded data was fully anonymized.

#### 3.2.2. EEG

For the EEG recording, we used the g.tec Nautilus mobile 16-channel EEG system. The recording was performed using the LSL (LabStreamingLayer) recorder software. The data was recorded at 500 Hz at the following electrode positions: CZ, FP2, F3, FZ, F4, FT7, C3, FP1, C4, FT8, P3, PZ, P4, PO7, PO8, OZ of the 10–20 system. Cz was used as a recording reference, with another reference on the right earlobe of the participant. All the recorded EEG data was filtered and re-referenced as processing. Impedances were kept below 20 k℧. The setup combined with the HoloLens can be seen in Figure 5A.

#### 3.2.3. Eye Tracking

Eye gaze was recorded using a binocular, wearable Pupil labs eye tracker at 120Hz, using the provided recording software. The eye tracker was fixed to the HoloLens, and distance and angle were adjusted to the eyes for every participant. The calibration is displayed on the HoloLens^1^: The participant has to focus at a point for 1 s (60 frames), and there are 9 points in total which are displayed on an ellipse matching the screen size of the HoloLens. The recordings will be used in further additional classification approaches. In the context of this paper, eye tracking data will not be analyzed further.

#### 3.2.4. Behavioral Data

All interaction data (especially head movement and current position) were recorded during the trial. Performed movement is evaluated for the accuracy assessment of the internal task. Also, the orientation of the sphere and the tube were tracked and their distance in the viewport. The later is used to measure the success of the alignment in both conditions. In total, we record the orientation of the ring, and tube for both the internal and external condition, the orientation of the head of the participant, and a measure of the alignment of the internal and external ring and tube during each trial. The streams are described in detail in section 6.

### 3.3. Analysis

In this section, we describe how we processed and analyzed the data collected by the LabStreamingLayer (LSL) to distinguish internal and external attention in a binary classification task. The processing scripts were written in Python 3.7 using the MNE toolbox, numpy, matplotlib, scikit-learn, pandas, and scipy. All analysis steps described were performed offline. The recorded LSL file was split into EEG and context features after synchronizing the marker stream with the rest of the data based on the timestamps, and both data collections were stored in the MNE data-format. Training and test data were split in a 80/20 manner and only the training data was used for normalization and hyperparameter optimization. The classifier that was trained on the training set in a 5-fold cross validation was assessed on the test set and those accuracies are reported in the results. With the future goal to later establish a real-time classification of internal and external attention, the preprocessing and manual evaluation of each dataset was kept to a minimum.

#### 3.3.1. Alignment and Task Performance

**Figure 7:**
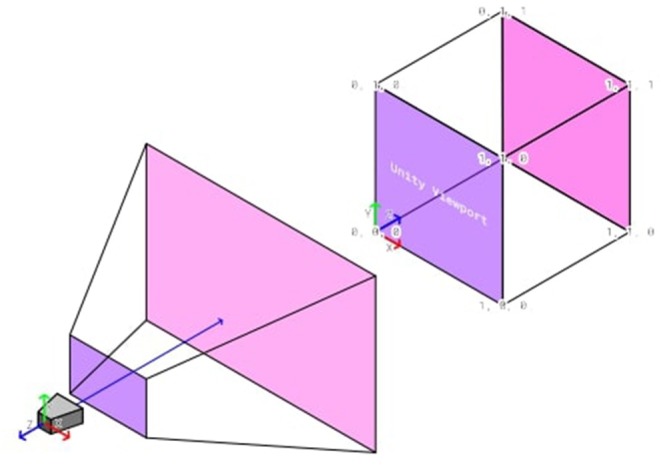
The transformation of the Unity camera space to the uniform Unity-viewport space.

For checking the misalignment, we make use of the render engine of Unity and the HoloLens. Since the experiment objects are virtually rendered objects which are displayed by the HoloLens close to the eyes of the participant, we can check if the objects align on the virtual camera in the experiment application. To do so, we can check the euclidean distance between the tube and the sphere's viewport coordinates. The viewport represents a normalized space in which 3D objects are transformed before an image can be rasterized by the render engine, as visualized in Figure 7. Only objects are processed further, which are located in the view frustum of the virtual camera. The viewport bottom-left relative from the camera is (0, 0), the top-right is (1, 1) for x and y-coordinates. For our alignment measurement, we can neglect the z-coordinates because those store only depth information which is not needed for the perspective alignment.

#### 3.3.2. EEG Preprocessing

Preliminary to a visual inspection, the EEG data was low-pass filtered at 50 Hz, high-pass filtered at 1 Hz, and an additional notch-filter was applied at 50 Hz to exclude all effects of the powerline-noise. During the inspection of the data, broken channels that were noted down during the experimental session were excluded, and later interpolated (an average of 2 channels were excluded). Afterward, we re-referenced the data to the average of all channels. The marker stream was then used to cut the data into epochs. Tutorial data was not considered and cut-off. Every epoch is a 13-s window in reference to the marker indicating the start of an internal or external trial. The epoch starts 1 s after trial onset (to exclude the effects of auditory cues) and has a duration of 13 s (to exclude the effect of different trial lengths in internal and external trials). The epochs are baseline-corrected based on the first second of each epoch.

A manual artifact removal was considered but with regard to a future real-time approach dismissed as not suitable for our purpose. An automatic artifact removal was tested by performing and Independent Component Analysis (ICA) on the data and rejecting eye movement of muscle related components. The improvement of the classification accuracy was marginal or not visible for most subjects. Thus, it was decided to perform no artifact cleaning on the datasets.

#### 3.3.3. Feature Extraction

We did not initially restrict the number of features but used statistical feature selection methods later to identify promising feature sets. Following previously described literature, we decided to extract EEG-features based on the Power Spectral Densities (PSD). We computed the power spectra using the multitaper method for the α (alpha, 8–14 hz), β (beta, 14–30 hz), θ (theta, 4–8 hz) and γ (gamma, 30–45 hz) -band and calculated the average and maximum power for each channel in each frequency band in each trial. This resulted in a feature vector containing *number of channels x 2 x 4* features. The frequency band ranges were chosen, based on Abo-Zahhad et al. (2015). All feature names mentioned in this paper follow the following naming convention: fmin+channel+[mean,max] with *fmin* being the lower bound of the frequency band.

Calculating more frequency bands by splitting the wide β-band into lower (14–22 hz) and upper (22–30 hz) β-bands or including the δ-band (1–4 hz) did not improve the preliminary classification results and was not considered further.

#### 3.3.4. Hyperparameter Optimization

**Figure 8:**
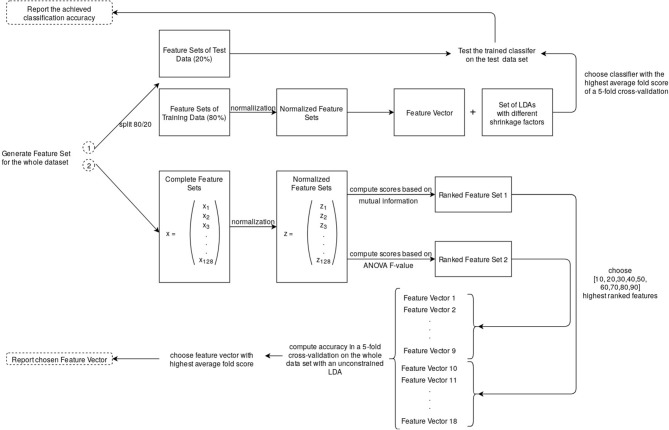
During the first pipeline: The feature vector was selected through a hyperparameter optimization in a 5-fold cross-validation on normalized features (16 channel recording → 128 features). See section 3.3.3 for the preceding steps that led to the feature set.

The pipeline that led to the reported results is displayed in Figure 8. We chose to normalize the feature values (x) by subtracting the mean (u) and scaling them to unit variance (s) before determining the number of best features individually for each participant or training the classifier.

$$\begin{array}{l} z=\frac{\left(x-u\right)}{s} \end{array}$$

We decided to run two versions of the classification pipeline. The first version included an individual feature selection and was used to estimate which combination of which features led to the best classification, and whether a selected feature set for all participants might improve the average classification accuracy (Figure 8, 2). The chosen features were also interpreted. The second pipeline was essentially the same, but without an automatic feature selection and an optimized shrinkage instead. The results of the second pipeline are the reported classification accuracies, whereas the first pipeline was used for the described analysis of the features and alternative feature sets for the individual classifiers. Feature selection included a hyperparameter optimization concerning score function and the number of selected features. In a grid search, the scoring was done by estimating the mutual information between each label and feature, as well as the ANOVA *F*-Value. The method, leading to a feature set that yields a higher accuracy, was selected. These feature sets were then evaluated in a 5-fold cross-validation by computing the average score of each fold and choosing the highest scoring number of features. The possible selection of features laid within a range of 10–90 with a step size of 10.

In a further step, we evaluated whether the classification accuracy for a participant with a classification accuracy below 80% would improve with a global feature set that is based on the features that were chosen during the classification of a participant with a high accuracy. We tested two different approaches to evaluate this hypothesis. The feature selection for this feature set was once based on the number of times a certain feature was chosen by the better half of the preliminary classification results and once by the average score the individual feature was assigned during the feature selection step. However, this global feature set did not lead to an improvement of the classification accuracies and will not be considered further.

In a second version of the classification pipeline, the whole feature set was used but combined with a constrained classifier (Figure 8, 1). The results of this approach are the reported accuracies in the results section 4.1.1. We first tried an approach, where we calculated the shrinkage factor using the Ledoit-Wolf lemma. This led to a lot worse results than an individual feature selection. Instead, we included the shrinkage level in the hyperparameter optimization by performing a grid search for the best shrinkage factor (0.1 to 0.9, step size = 0.1). A combination of feature selection and a constrained classifier did no further improvement to the classification accuracy. Thus, for the final results, classifier with an individual shrinkage factor was chosen to be trained on the training data feature set and tested on the test data.

#### 3.3.5. EEG Classification

Wang et al. (2007) argue that a Linear Discriminant Analysis (LDA) is very well suited for binary classification tasks. In this paper, the focus lays on proving a simple but accurate EEG-data classification for AR rather than optimizing classification results. Thus, we chose LDA as our classification approach without further comparisons to other algorithms. The LDA was performed with shrinkage and with a least-squares solver. Training was performed on 80% of the trials. The split into training and test data was stratified and not shuffled. The final score was calculated on the test set (see Figure 8). The scikit-learn toolkit was used for feature selection, parameter optimization, cross-validation, and the classifier.

## 4. Results

### 4.1. EEG Classification

The main goal of this research was to classify EEG trial data based on internal and external attention. This was pursued by the analysis steps described in section 3.3. Apart from the achieved classification accuracy, the features chosen by the automatic feature selection are of importance for the interpretation of the results.

#### 4.1.1. Classification Accuracy

The main quantitative results of this study are presented in Table 1. The score was computed for all participants in a balanced binary classification task with a chance level of 50%. The accuracy is calculated, based on how many predicted labels were in accordance with the original label. Overall highest achieved classification accuracy was 100% for 3 participants, and 6 of 14 participants' data was classified with an accuracy of more than 90%. All but one participant's data resulted in a score higher than 70%. The average classification accuracy was 85.37% ± 11.27%.

**Table 1:** The classification accuracy for each subject.

**Participant**	**EEG-classification accuracy (%)**	**Movement-classification accuracy (%)**
1	66.67	49.11
2	66.67	63.57
3^*^	73.33	89.11°
4	80	61.07
5	80	86°
6	80	90.54°
7^*^	81.82	74.67
8	86.67	67.5
9	93.33	86.07
10	93.33	90.54
11	93.33	94.46°
12	100	68.21
13	100	76.61
14	100	82.14
Average	85.37	77.08
Variance	11.27	13.53
Chance	50	50

Sorted by EEG-classification accuracy. Average and variance are computed on unrounded results (*participants with fewer trials due to technical problems, ° better classification accuracy than for the EEG classification).

#### 4.1.2. Features

An examination of the selected features from the better scoring participants (accuracy > 80%), as described in Chapter 3.3.4, revealed that 53.25% of the features were generated from frontal channels. Features chosen by less than half of the participants with the appropriate accuracy were excluded for this analysis. In comparison, only 7 of the 16 electrodes are placed in frontal positions. Thus, an equal distribution of selected features would include 43.75% frontal features, and our analysis shows the importance of frontal features for attention classification.

Separating the features based on the frequency band they were derived from yields 18.77% β-band, 29.88% θ-band, 21.83% α-band, and 31.8% γ-band features as selected features. In the complete feature set, all frequency bands are equally distributed (25% of the features were calculated based on each feature band).

### 4.2. Task Performance Analysis

For analyzing the task performance, we looked at the alignment values and the head movement per participants in order to find a correlation with the EEG-classification results.

#### 4.2.1. Alignment

**Figure 9:**
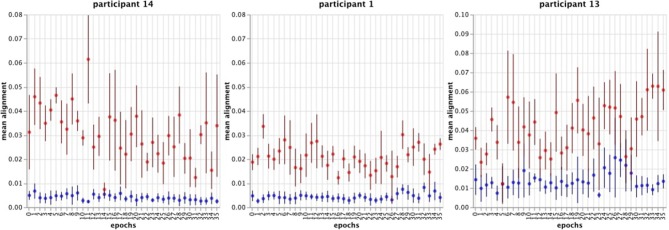
The graphs show the mean alignment for participant 14, 1, and 13. The blue points mark the external condition, red the internal. The error bars denote the standard deviation (±) for a trial.

For each participant, we calculated for each trial the mean and standard deviation of the alignment values recorded during the experiment for both conditions. Figure 9 displays the results for participants 14, 1 and 13. Then we compared the internal and external condition per participant with a two-sided *t*-test for two independent samples (α = 0.05). For each participant, the alignment differs significantly (mean comparison: highest *p*-value = 0.00015; standard deviation comparison: highest *p*-value = 0.0033).

#### 4.2.2. Head Movement

**Figure 10:**
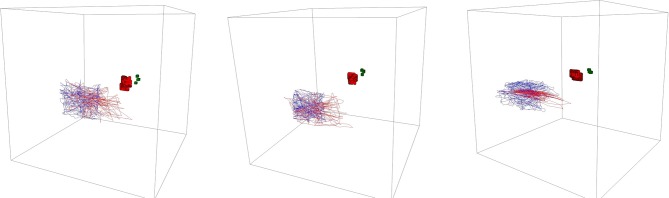
3D plot of the head movement of participant 14, 1, and 13 (from left to right). The blue lines denote the external condition, red the internal. In the background, one can see the visible constellation (red overlying spheres and green overlying boxes) and hidden constellation (overlying turquoise spheres and purple overlying boxes).

To get a better understanding of what the participant's movement, we generated 3D plots for the head movement for each participant. Figure 10 shows three examples. The main observation is that most of the participants have a close alignment with the visible constellation during the external task whereas they diverge more during the internal condition – their movement is wider but still shows a similar pattern compared to the movement of the hidden constellation in case the task was performed correctly (see participant 13 in Figure 10 as an example for a not well-performed movement).

**Figure 11:**
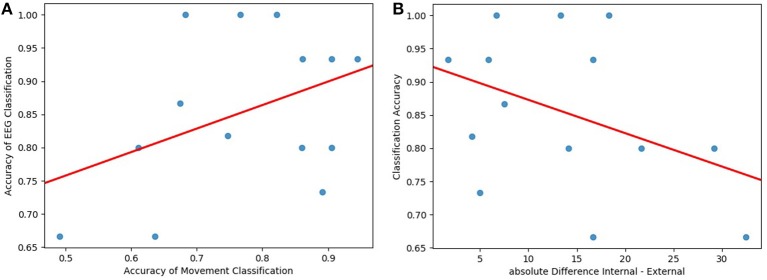
**(A)** The classification accuracies from the movement data correlate weakly with the classification accuracy (*Pearson's r* = 0.41, visualized by the red line). **(B)** The task load assessment differences between the internal and the external condition correlate negatively with the classification accuracy (*Pearson's r* = −0.4, considered weak, visualized by the red line).

Furthermore, we analyzed the speed of the head movement. For each participant and each trial, we calculated the mean, the standard deviation, median, minimum, and maximum speed value. Based on this data, we trained a Linear Discriminant Analysis model with 5-fold cross-validation to identify whether we can classify the conditions robustly by just comparing the speeds of the head movement. In Table 1, we present our results. These classification accuracies were compared with the classification accuracy of the EEG data for each participant. The classification accuracies do not show a measurable correlation with Pearson's Correlation *r* = 0.41 (see Figure 11A).

### 4.3. Questionnaires

To further ensure that the measured and decoded differences rely solely on internal and external attention, we analyzed the collected questionnaires in relation to the classification results. Furthermore, noticeable questionnaire results could explain outliers.

#### 4.3.1. Task Load Analysis

The NASA Task Load assessment was adapted for our purposes and did not contain weighted categories. All answers were weighted equally and translated to a scale from 0 to 100 (100 = high load). Table 2 summarizes the results averaged over participants. The categories *Effort* and *Physical* were not rated significantly higher concerning task load for the internal or external task. For all other categories, the internal task load was rated significantly higher (*p* < 0.05). Only the difference in ratings for the *Mental* category was highly significant with a higher task load for the internal task (*p* < 0.001).

**Table 2:** Results of the NASA Task Load assessment averaged over participants (◇ significant, ◇◇ highly significant).

**Demand**	**External task**	**Internal task**	***p*-value**
Effort	54.50	68.17	0.055
Frustration	34.17	59.17	0.007◇
Mental	27.83	68.00	0.00043◇◇
Performance	67.50	46.67	0.02◇
Physical	58.17	54.67	0.5
Temporal	21.17	36.50	0.03◇
Mean	37.83	52.33	0.0013◇
Variance	23.57	22.39	not tested

To estimate possible interferences with the difficulty differences of the tasks to classify internal and external attention, we computed the correlation between the classification accuracy and the absolute difference in task load rating for each participant. The overall external rating was subtracted from the overall internal rating. Concluding, a higher difference score signifies that the participant perceived the internal task as much harder while a negative score would imply that the participant perceived the external task as harder. Since the goal is to compare task load differences, the absolute difference was taken (For completeness: Two subjects rated the external task load as harder). Figure 11B shows the relationship between accuracy and task load rating difference. We calculated Pearson's r as a measure for the correlation between the variables. The resulting correlation of *r* = −0.4 is a moderate negative correlation. The data of participants who rated the task load difference as higher was decoded worse by our classifier.

#### 4.3.2. Mind Wandering Questionnaire

The answers of the mind wandering questionnaire were evaluated and related to the results of the classification (see section 4.1.1) and the Task Load Analysis (see section 4.3.1). However, no relation or significant differences were found, and thus, the results will not be reported further.

## 5. Discussion

In this study, we tested the possibility to classify internal and external attention in an AR setting with machine learning techniques. To provide an appropriate paradigm that includes two different task versions (one for internal and one for external attention), we implemented a spatial alignment task on the HoloLens. The two versions of the task were identical, except for the direction of attention. The visually displayed scene in the external task had to be imagined for the internal task, leading to the same movement patterns and cognitive tasks. The analysis of the movement data showed that the tasks were performed correctly and understood well by the participants. The slightly higher misalignment between the target and the object in the internal task was expected and hard to avoid because of missing visual feedback. However, the overall performance for the internal task of every participant was satisfactory, and we can claim that all participants understood the task and tried to perform it correctly. The NASA Task Load Index evaluation stressed that the main task difference was the mental component, as desired. The correlation analysis of the task load assessment and the classification accuracy proved that the classification did not improve for participants that assigned a much higher load to internal tasks. In conclusion, the classifier was not trained to differentiate the trials based on the task load.

The same analysis was performed to exclude the possibility that we are classifying movement patterns in the EEG data. The comparison of the classification accuracy based on movement data and the classification accuracy based on EEG data showed no correlation between the two classification results. Hence, the classifier was not trained to differentiate the trials based on the movement of the participant.

During the internal task, the AR objects were still present, but not moving. This way we ensured that the classified difference is not because of the presence of visual AR input or feedback. However, the imagery component of the internal attention exercise is exclusive to the internal task and not present in the external task. One could argue that the classified difference is “imagery” vs. “no imagery.” There is no task that is defined as general internal attention but internal attention has many facets and imagery is one of them that is resent in many tasks that involve internal attention. Therefor, we generalize here and future tasks will focus on different facets of attention to prove our findings.

After excluding task load and movement, we conclude that our classifier classified internal and external attention variations. Further arguments for this will be given in the following.

The results of the classification suggest that reliable decoding of the attentional state in this setting is possible. Without many optimizations of the classifier or the feature selection, the achieved classification accuracy was higher than chance level for all the participants. Half of the classification accuracies were above 85% with an LDA on almost unprocessed EEG data. The exclusion and interpolation of a few channels were necessary due to a broken electrode cap. Additionally, only simple filtering, baseline corrections, and re-referencing were performed. A preprocessing step that does not require the visual inspection or specialized cleaning steps is favored in consideration of a possible real-time classification.

The substantial differences in classification accuracy between the subjects could have multiple reasons. As expected, the internal task was rated with a higher task load and thus, perceived as more complicated than the external task. Informal feedback of the participants after the experiment also suggests that the task was hard to perform. However, as mentioned, the option of a misunderstood task or bad alignment performance due to inadequate internal attention or “mind-wandering” and a low level of concentration has been excluded by the analysis of the movement and alignment data. We found no correlation between subjects that had higher misalignment variations in the internal or external task and the according accuracy of our classifier.

One approach to improve the classification accuracy for the worst participant was to inspect the EEG data for artifacts visually. Epochs that included very noisy data were excluded, and an ICA was performed to identify eyeblink artifacts that were very present in the signal of all frontal electrodes. These artifacts were not as severe in any other participants' data. Supervised data inspection for artifacts and ICA cleaning resulted in better data that was classified with an accuracy of 70.3%. In the scope of this work, such intense manual preprocessing is not desired, and therefore, these results will not be considered.

Another possible explanation for lower classification accuracies could be a change over time. This concerns two modalities: firstly, the participant could experience a training effect and perform the internal task with ease after several trials, leading to a decreased need for internal attention. Secondly, the combination of the HoloLens and the EEG-cap on the head of the participant was prone for slight movements and changes. As a result, the data quality often decreased over time, and later trials had worse data than earlier trials. Our approach to reduce these effects as much as possible was choosing a stratified 5-fold cross-validation. Even with this approach, decreasing attention and increasing signal noise can worsen the classification accuracy.

The literature suggests person-dependent frequency bands (Newson and Thiagarajan, 2019). The predefined frequency band boundaries during the feature extraction could favor some participants. This could be solved with smaller bins to calculate the PSD on but would result in a broader feature set and longer classification times. Our attempt to split the β-band did not lead to an overall improvement of the classification results but individually chosen, or more fine-grained frequency bins could improve the classification.

A further reason that we can not exclude in retrospective or test is BCI illiteracy (Thompson, 2018).

Another result that supports our statements that we classify the trials based on attention-related differences and that better data quality would improve the classification accuracy is the evaluation of the selected features. The analyzed features suggest that the most critical information for the classification can be found in frontal electrodes and within the γ and α-bands. This is in accordance with the literature concerning attention (Cooper et al., 2003; Braboszcz and Delorme, 2011; Chun et al., 2011; Benedek et al., 2014). On top of that, the importance of these electrodes explains the lower classification accuracy of some participants because those electrodes were often disturbed by the setup with the HoloLens and one frontotemporal electrode was broken in many experiments. In the preprocessing of the data of participant 1 (who had the lowest classification accuracy), FZ, FP1, and FP2 had to be excluded and interpolated. Reducing these sources for noise could improve the classification. For participants for whom the accuracy was high, the selected features were very similar, whereas the automatic feature selection of the other participants chose different features. The recorded data for the better features was probably too noisy, and they were not chosen.

In the future, the recorded data will be analyzed and classified with regard to multiple modalities to integrate the eye tracking data and improve the classification accuracies. Furthermore, the results will be integrated into a real-time adaptive AR application and checked in further experiments. Additionally, more participants could be recorded with additional EOG and EMG electrodes for an automatic detection of eye and muscle artifacts. These tests will dispel more doubts about the purposeful task at hand and its results.

## 6. Conclusions

We showed that the classification of EEG data that was recorded in an AR paradigm based on internal and external attention is possible. The novel paradigm invented for this purpose seems to be very suitable. Even with simple machine learning principles and basic preprocessing steps, the classifier was able to reliably predict the attentional state of the participant in the offline analysis with perfect accuracy in 3 participants. None of our tests supports the assumption that anything but the actual attentional state was classified. The differences between the participants might be reducible. A future real-time system and multi-modal classifier are to be implemented.

## Data Availability Statement

The datasets generated and analyzed for this study, as well as the task, can be found online under https://osf.io/avwc2/.

## Ethics Statement

The studies involving human participants were reviewed and approved by Ethics Committee of the University of Bremen. The participants provided their written informed consent to participate in this study.

## Author Contributions

L-MV, FK, and FP contributed conception and design of the study. L-MV organized the database, performed the statistical analysis of the questionnaires and EEG data, wrote the first draft, and sections of the manuscript. FK implemented the experiment, performed the statistical analysis of performance and movement data, and wrote sections of the manuscript. L-MV and FK recorded the participants. All authors contributed to manuscript revision, read and approved the submitted version.

### Conflict of Interest

The authors declare that the research was conducted in the absence of any commercial or financial relationships that could be construed as a potential conflict of interest.

## Figures and Tables

**Figure A1:**
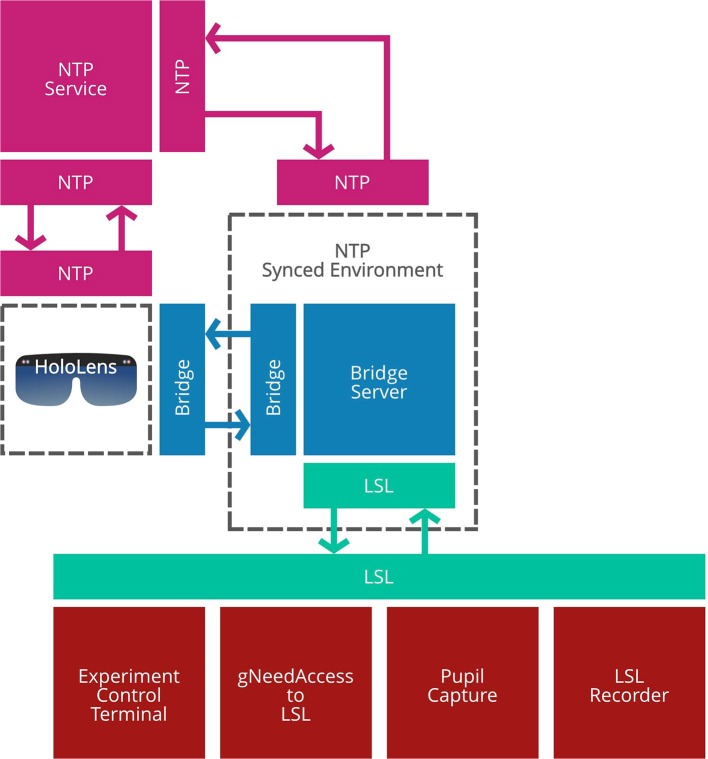
The experiment architecture is based on LSL communication platform. The graphic gives an overview of the different components involved in time-synchronous data exchange.

## Footnotes

^1^Pupil Labs provides a library for Unity – https://github.com/pupil-labs/hmd-eyes

## Appendix

### Implementation

The spatial perspective alignment task was build with the Unity engine^2^ and the HoloToolkit^3^. For network communication and recording of all data sources, we used Lab Streaming Layer (LSL)^4^. It is the backbone of the experiment architecture, as visualized in Figure A1. LSL is not supported on the HoloLens operating system, and therefore we provide a Transmission Control Protocol (TCP) based Client-Server bridge service which as capable of passing forward and backward data between LSL and the HoloLens. To ensure that the time of the HoloLens is synchronized with the time of the bridge server's operating system, we synchronize both systems with a Network Time Protocol (NTP) service^5^. Furthermore, we define and record multiple data streams from LSL. The following list provides information about their content:

- *Control stream*:
  send control-strings to the HoloLens to control the states of the experiment.
- *Control-return stream*:
  contains string-answers when a control-command was received by the HoloLens successfully. The content of an answer is whether the received control command is valid or not.
- *Marker stream*:
  Contains short-typed markers. A marker ranges from 0 to 9. The numbers represent the following meanings – 0, start of experiment app; 1, beginning of an experiment block; 2, end of an external trial; 3, start of the announcement of grid-numbers; 4, end of the announcement of grid-numbers; 5, start of the internal trial; 6, end of the internal trial; 7, start of the external trial; 8, end of an experiment block; 9, closing of the experiment application.
- *Head-orientation stream*:
  Contains the 4 × 4 orientation matrix of the head in virtual-coordinates as a float 32 array that is generated from quaternion and three-dimensional position vector in Unity World-Space.
- *Sphere-orientation streams for the hidden and visible sphere*:
  Same data format as the head-orientation stream.
- *Tube-orientation streams for the hidden and visible tube*:
  Same data format as the head-orientation stream.
- *Grid-number streams for the hidden and visible constellation*:
  this stream contains the grid-number to which the constellation will rotate. The value type is “short.”
- *Visibility streams for the hidden and visible constellation*:
  We stream the visibility of the constellation, meaning whether or not the participant sees the objects on the HoloLens displays. This is required because the HoloLens has a small field of view. If the sphere and tube are both not visible simultaneously a “0” is sent, else a “1.” The value type is “short.”
- *Alignment-measure streams for the hidden and visible constellation*:
  We send a float32 value that represents how well the participant aligns the sphere and the tube (see section 3.3.1 for more details). It is important to do this for the visible constellation and the invisible one.

The implementation of the task is open-source and accessible from https://gitlab.csl.uni-bremen.de/fkroll/ieaExperiment.
